# Supplementary material for: Protease-activation using anti-idiotypic masks enables tumor specificity of a folate receptor 1-T cell bispecific antibody
Source: Nat Commun. 2020 Jun 24;11:3196. doi: 10.1038/s41467-020-16838-w (PMC7314773; doi:10.1038/s41467-020-16838-w)
Supplement: Supplementary file 1 — Supplementary Information [file 41467_2020_16838_MOESM1_ESM.pdf]

# Protease-activation using anti-idiotypic masks enables tumor specificity of a folate receptor 1-T cell bispecific antibody

Geiger *et al.*

## Supplementary Figures

Supplementary Figure 1

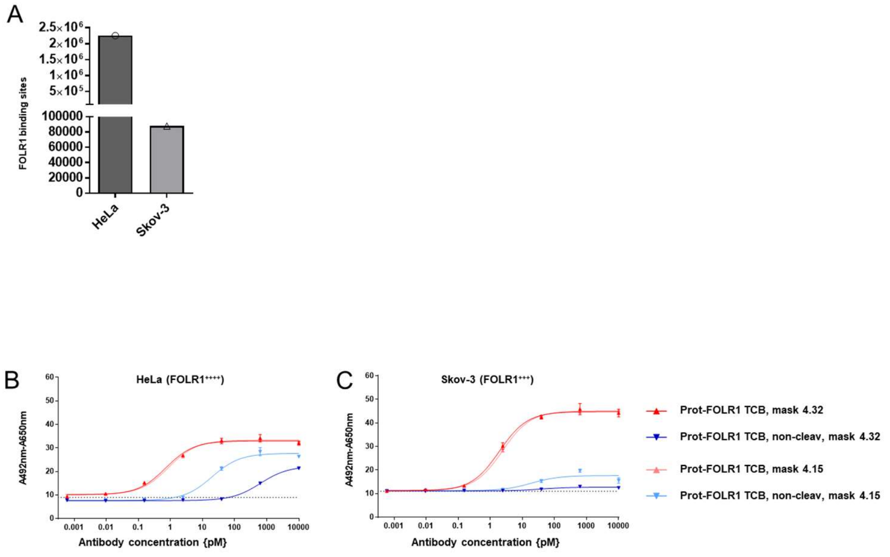


**Supplementary Figure 1 Masking efficiency is dependent on target cell expression level and affinity of mask**

(**A**) FOLR1 binding sites were calculated for the different cell lines. Each bar represents the value calculated for the indicated cell line. Antigen binding sites were calculated using calibration beads and technical triplicates (n=1). (**B** and **C**) Dose–response curves for T-cell killing of FOLR1 positive tumor cells after 48 h mediated by TCB using PBMCs as effector cells with an effector to target ratio of 10:1. Comparison of Prot-FOLR1-TCB with two different masks called 4.15 and 4.32 (non-cleav and pre-cleaved with matA site) for tumor cells with different FOLR1 expression levels (**B** HeLa cells, **C** Skov-3 cells). The cytotoxicity induced by the TCBs is shown. Each point represents the mean of triplicates. Standard deviation is indicated by error bars (representative experiment for one PBMC donor, n = 2 different human PBMC donors). For the calculation of EC50 values a sigmoidal dose-dependency was calculated (GraphPad Prism 6).

Supplementary Figure 2

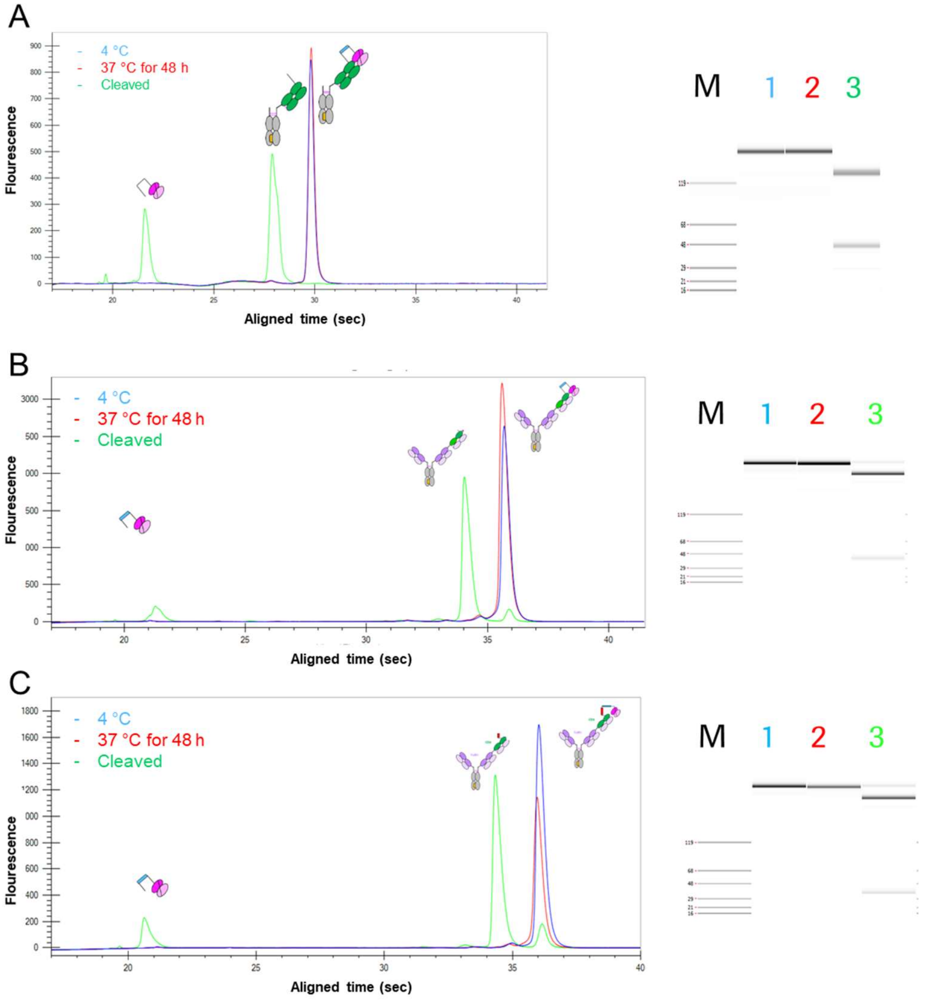


**Supplementary Figure 2 Protease-activated antibodies can be cleaved by recombinant matriptase**

Capillary electrophoresis (CE-SDS) (non-reducing conditions) and electropherogram showing either the untreated antibody stored both at 4 °C (blue) and at 37 °C (red) for 48 h or the pre-cleaved antibody (green). (**A**) Prot-mαCD3 IgG, matA site. (**B**) Prot-FOLR1-TCB, matA site. (**C**) Prot-FOLR1-TCB, MMP-matA site.

## Supplementary Figure 3

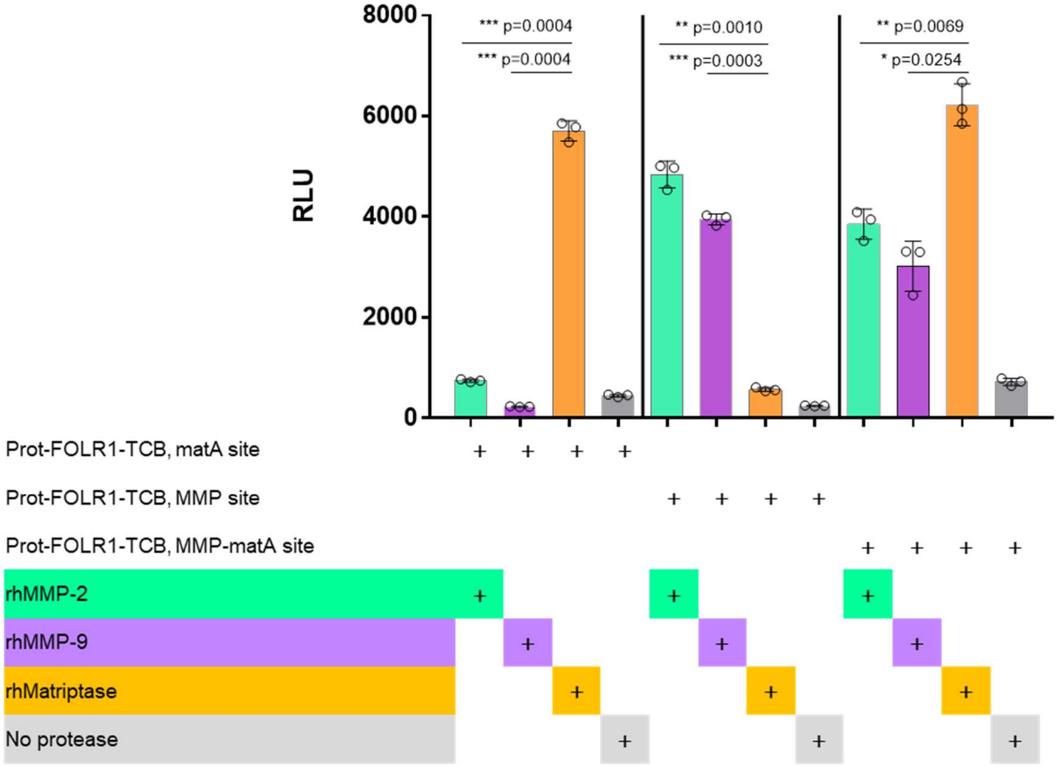


**Supplementary Figure 3 Specific cleavage of recombinant human MMP-2, MMP-9 and matriptase**

Cleavage of matA, MMP or MMP-matA linkers by rhMMP-2, rhMMP-9 and rhMatriptase was analyzed. Prot-FOLR1-TCBs were bound to plate coated anti-human Fc antibody after incubation with recombinant protease. A Jurkat NFAT reporter assay was used to check cleavage of linker by recombinant MMP-2, MMP-9 or matriptase. Prot-FOLR1-TCBs with different cleavage sites (MMP, matA or MMP-matA) were incubated either with rhMMP-2, rhMMP-9 or rhMatriptase before they were bound to plate coated anti-human Fc antibody. Jurkat NFAT reporter assay was used to check linker cleavage by measuring relative

luminescence units (RLU) depending on CD3 binding. Each bar represents the mean value of technical triplicates, standard deviation is indicated by error bars (representative experiment of n = 2). For statistical analysis two-tailed, paired t-test was used. P values are included in the figure.

Supplementary Figure 4

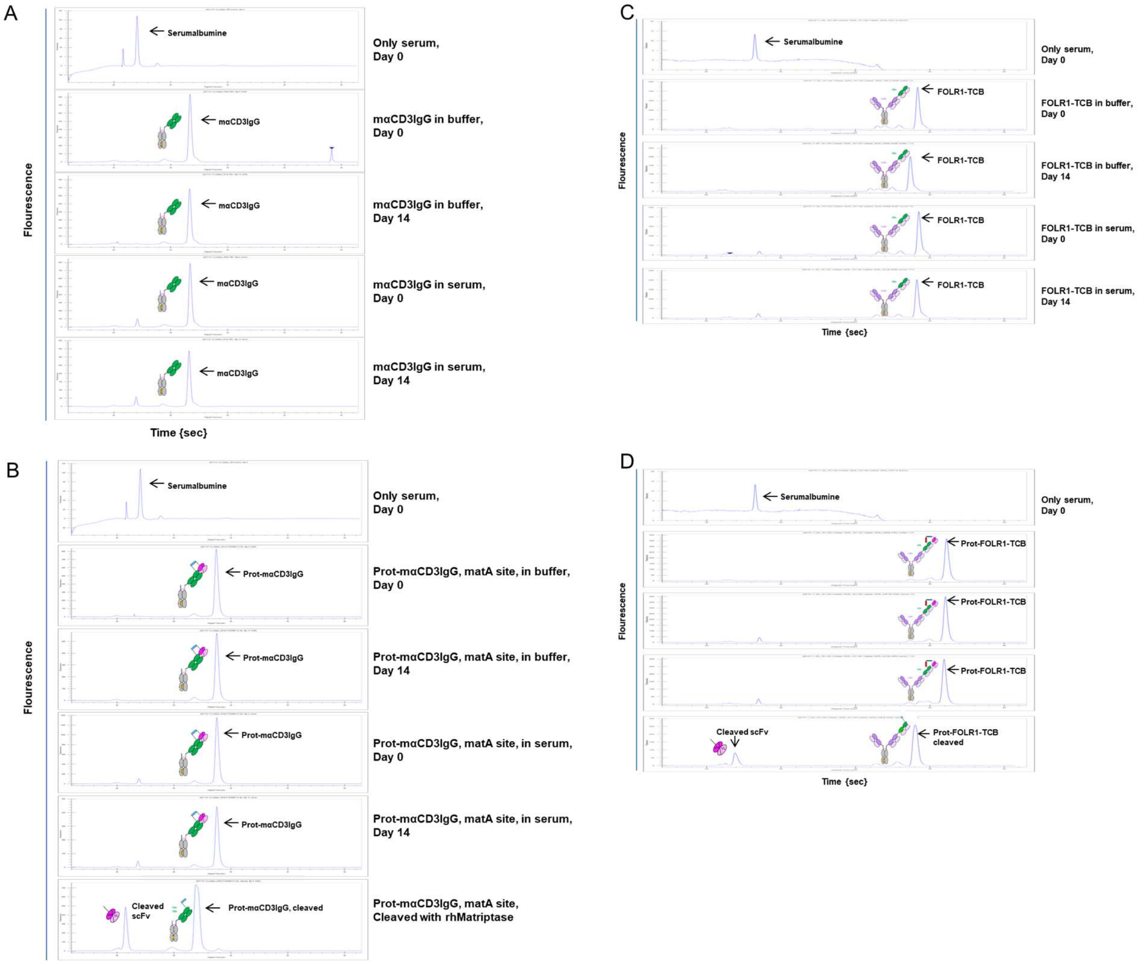


**Supplementary Figure 4 Integrity of protease-activated antibodies was retained after incubation in human serum**

Capillary electrophoresis at non-reducing conditions for (**A**) Prot-mαCD3 IgG (**B**) Prot-mαCD3 IgG, 4.32, matA site (**C**) FOLR1-TCB (**D**) Prot-FOLR1-TCB, MMP-matA site.

CE-SDS analysis of antibodies after incubation in human IgG depleted serum for 14 days at 37 °C. The antibodies were purified via affinity chromatography using Protein A HPLC before the integrity (capillary electrophoresis at non-reducing conditions) was analyzed (n=1).

## Supplementary Figure 5

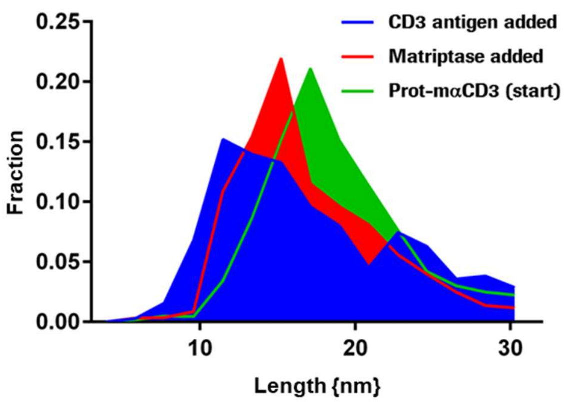


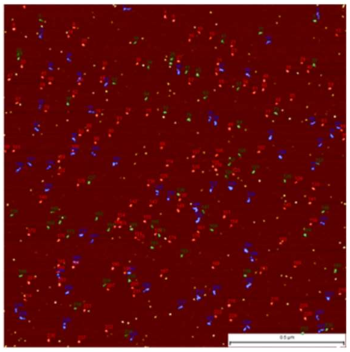

| Class Name | Count | Percent [%] | Density [1/nm²] | Shape Coverage [%] | Coverage [%] |
|---|---|---|---|---|---|
| Antigen (n.D.) | 0 | 0.00 | 0.00 | 0.00 | 0.00 |
| cleaved | 121 | 51.05 | 0.00 | 37.06 | 0.49 |
| masked | 55 | 23.21 | 0.00 | 22.50 | 0.30 |
| complexed | 61 | 25.74 | 0.00 | 40.43 | 0.53 |

**Supplementary Figure 5 CD3 Fab can bind to CD3 antigen upon linker-cleavage**

The AFM data shown in Figure 3 demonstrate the de-masking and complexation step on the level of individual molecules. The particles observed with AFM match in respect to their length the class averages resulting from multivariate statistical analysis of NS-TEM micrographs. The EM-AFM data complement each other and confirm the structural-functional validity of the demasking complexation concept on a qualitative and structural level.

The AFM method enables to monitor individual molecules which reversibly adhere on surface of mica and in solution, at functional states. In the beginning of the experiment a homogenous population of masked molecules (green histogram) is observed. After addition of matriptase the length histogram is changing and a population of slightly shorter molecules rises (red histogram). The addition of the Fc-CD3 antigen let appear two additional populations (blue

histogram), of most small particles with a length corresponding to the free antigen, as well as particles which have a length expected for the large complex. The AFM micrograph at the top right was recorded during the experiment, and at a time point after the decorated surface has been exposed to Matriptase and the CD3 antigen. The reaction is indicated to be not complete because a mixture of differently sized particles can be observed. The different particles are sorted by their size, as shown by different colors. Green particles corresponds to the masked construct, the red particles with de-masked particles, and the blue particles correspond to the complex, and the table at the bottom shows the numeric composition of the fractions mentioned above. The experiment has been conducted in the nanomolar concentration range to avoid monolayer formation on the surface of mica. The concentration of all educts and matriptase have been conditioned to enable stable imaging of the process with AFM, the particles are trapped on a surface and the data do not enlight the kinetic of the de-masking complexation reaction. Representative data shown (n=3 experiments).

Supplementary Figure 6

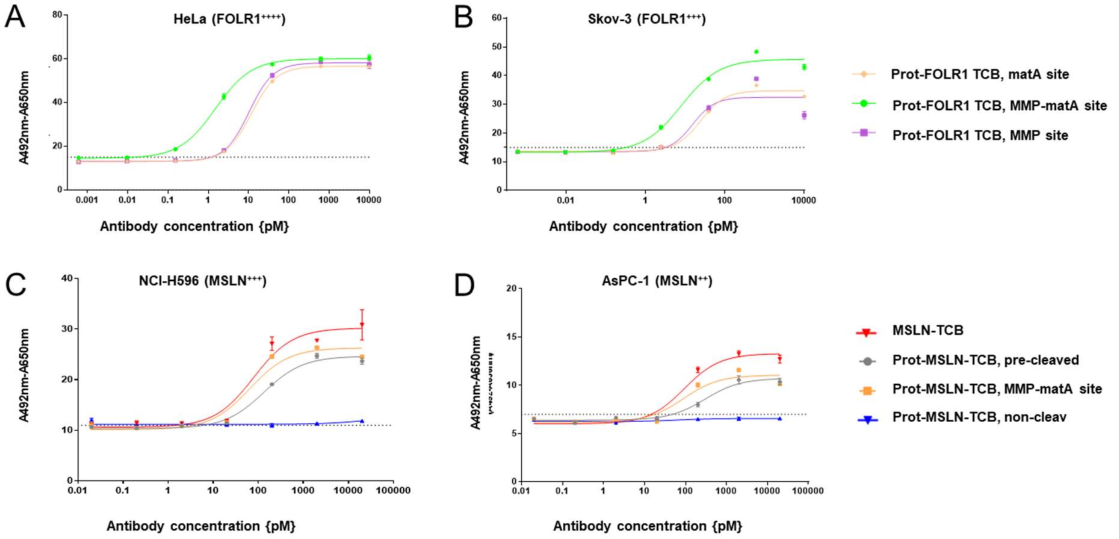


**Supplementary Figure 6 More effective activation of Prot-FOLR1-TCB by combination of cleavage sites and CD3-masking moiety is applicable for other TCBs**

Dose–response curves for T-cell killing of FOLR1 positive tumor cells after 48 h mediated by TCB using PBMCs as effector cells with an effector to target ratio of 10:1.

(**A** and **B**) Comparison of Prot-FOLR1-TCB with three different linkers (matA, MMP and combination of MMP-matA) using tumor cells (**A** HeLa cells, **B** Skov-3 cells). The cytotoxicity induced by the antibodies is shown. (**C** and **D**) The cytotoxicity induced by the Prot-MSLN-TCB containing MMP-matA site for two cell lines (**C** NCIH596 cells, **D** AsPC-1 cells) with different MSLN expression levels is shown. The Prot-MSLN-TCB (grey circles) or a non-cleav site (blue triangles pointing up) is shown. MSLN-TCB (red triangles, pointing down), pre-cleaved Prot- MSLN-TCB (orange squares) and a non-targeted TCB (black circles) are used as controls. The pre-treatment of the Prot-MSLN-TCB (orange squares) was done by incubation of Prot-MSLN-TCB with recombinant human matriptase for 24 h at 37 °C. Each point

represents the mean of technical triplicates. Standard deviation is indicated by error bars (representative experiment for one PBMC donor, n = 3 different human PBMC donors). For the calculation of EC50 values a sigmoidal dose-dependency was calculated (GraphPad Prism 6).

Supplementary Figure 7

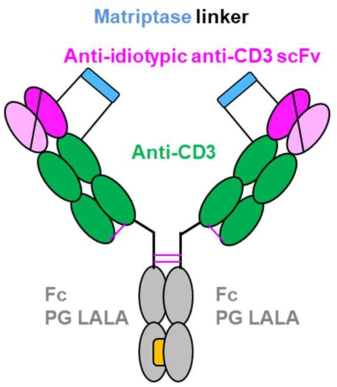


**Supplementary Figure 7 Design of bivalent Prot-αCD3 IgG with two anti-idiotypic anti-CD3 scFvs**

Design of protease-activated bivalent anti-CD3 IgG with two anti-idiotypic anti-CD3 scFvs fused to the N-terminus by a linker containing a matA cleavage site.

Supplementary Figure 8

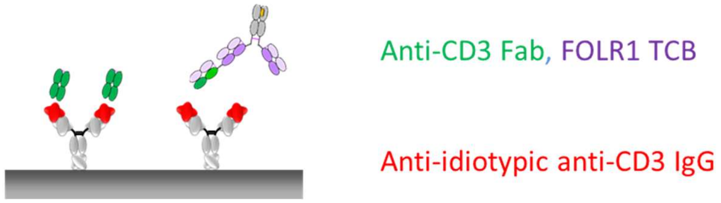


**Supplementary Figure 8 FOLR1-TCB was used to determine affinity of anti-idiotypic anti-CD3 IgG to CD3 binder**

Surface plasmon resonance measurements of anti-idiotypic anti-CD3 antibodies to anti-CD3 Fab and anti-CD3 in TCB format.

The anti-idiotypic anti-CD3 antibody was chemically immobilized. The Fab fragments of the anti-CD3 antibody (generated by plasmin digestion and purification) or the FOLR1-TCB were used as the analyte.

Supplementary Figure 9

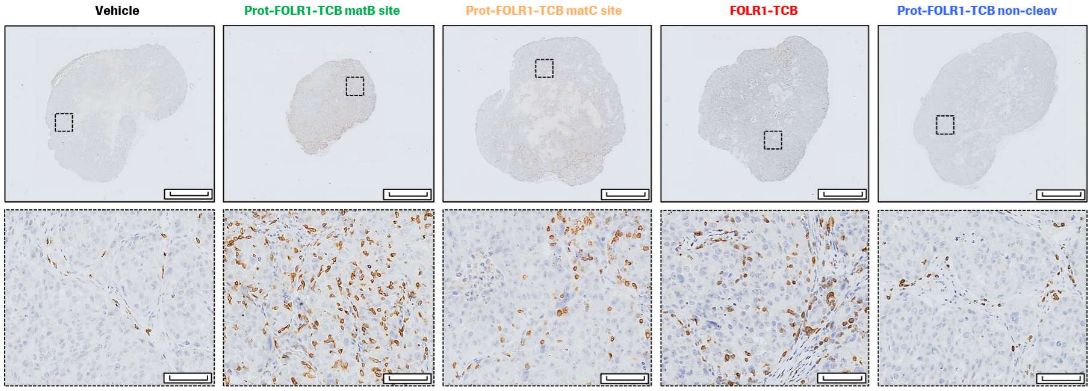


**Supplementary Figure 9 Significantly more huCD3 T cells were detected in Prot-FOLR1-TCB and FOLR1-TCB group compared to vehicle**

Representative images for huCD3 T cell count in tumors of the different treatment groups of in vivo efficacy study Figure 9A,B. At study termination, mice were sacrificed and tumors were surgically removed from all animals. CD3 stainings were performed in the Leica autostaine following the manufacture's protocols. Sections (4µm FFPE slides) were counterstained with hematoxylin (Sigma Aldrich) and slides were scanned using Olympus VS120-L100 Virtual Slide Microscope scanner. Scale bars are shown in the images. Scale bars indicate 4 mm (upper panel) and 100 µm (lower panel). Positive staining is observed as a brown precipitate within the sections. CD3 staining in tumor samples from the efficacy study have been performed on one section per tumor sample and 6-8 tumors / group were used.

Supplementary Figure 10

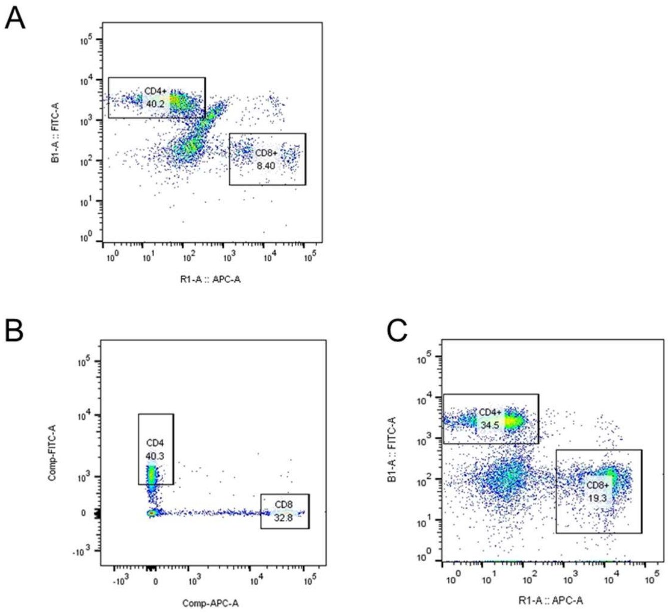


**Supplementary Figure 10 FACS gating strategies used for T cell activation measurements**

Gating strategy to identify $CD8^{+}$ T cells. Relative percentages for the gates are presented on the contour plot. CD4 positive cells were stained with CD4-FITC antibody, CD8-positive cells were stained with CD8-APC antibody. APC positive cell population was gated as CD8 T cells. Median fluorescence intensity for CD69 was plotted for CD8 positive cells.

(A) FACS gating strategy used in Figure 4B and D. (B) FACS gating strategy used in Figure 6 C. (C) FACS gating strategy used in Figure 6 D.

## Supplementary Tables

### Supplementary Table 1 Affinities of different anti-idiotypic anti-CD3 antibodies to anti-CD3 Fab is higher than in TCB format

SPR analysis of the anti-idiotypic anti-CD3 antibodies 4.15 and 4.32 against anti-CD3 Fab and anti-CD3 in TCB format. The mean equilibrium dissociation constant of two independent runs are shown.

| Clone ID | Anti-CD3 Fab | Anti-CD3 in TCB |
|---|---|---|
| 4.15 | 2nM | 20nM |
| 4.32 | 2 nM | 10 nM |

### Supplementary Table 2 Amino acid sequence of linkers (cleavage sites are underlined)

| Full name | Abbreviation | Amino acid sequence | Source for cleavage site | Linker length |
|---|---|---|---|---|
| MMP-2, -9-Matriptase A linker | MMP-matA | GGGGSVHMPLGFLGPRQARVVNGGGGGSGGGGS | | 33 |
| Matriptase A linker | matA | GGGGSGGGGSRQARVVNGGGGGSGGGGSGGGGS | | 33 |
| MMP-2, -9 linker | MMP | GGGGSGGGGSVHMPLGFLGPGGGGSGGGGSGGS | matC patent US10261083B2 | 33 |
| Non-cleavable linker | non-cleav | GGGGSGGGGSGGGGSGGGGGGGSGGGGSGGGGS | | 33 |

| | | | |
|---|---|---|---|
| Matriptase B linker | matB | GGGGSGGGGSPMAKKGGGGSGGGGSGGGGSGGS | 33 |
| | | 1 | |
| MatriptaseC linker | matC | GGGGSGGGGSLSGRSDNHGGGGSGGGGSGGGGS | 33 |

**Supplementary Table 3 All antibodies could be purified with less than 5 % impurities**

Monomer content of antibodies was analyzed by analytical size exclusion chromatography.

| Antibody | High molecular weigth impurities | Correct antibody | Low molecular weigth impurities |
|---|---|---|---|
| mαCD3 IgG | 0 | 100 | 0 |
| Prot-mαCD3 IgG, matA site | 1.3 | 96.9 | 1.8 |
| Prot-mαCD3 IgG, non-cleav | 4.5 | 95.5 | 0 |
| FOLR1-TCB | 0 | 100 | 0 |
| Prot-FOLR1-TCB, matA site | 3.3 | 95.6 | 1.1 |
| Prot-FOLR1-TCB, MMP-matA site | 0 | 100 | 0 |
| Prot-FOLR1-TCB, non-cleav | 3.4 | 96.1 | 0 |
| Prot-FOLR1-TCB, MMP site | 2.8 | 97.2 | 0 |
| MSLN-TCB | 2.0 | 96.2 | 1.8 |
| Prot-MSLN-TCB, MMP-matA site | 4.0 | 96.0 | 0 |
| Prot-MSLN-TCB, non-cleav site | 2.1 | 97.9 | 0 |

**Supplementary Table 4 Prot-mαCD3 IgG and Prot-FOLR1-TCBs with different linkers were thermaly stable**

Thermal stability of Prot-mαCD3 IgG and Prot-FOLR1-TCBs with different linkers. Temperature ranged from 25 °C to 85 °C with a heating rate of 0.1 °C / minute. The aggregation onset temperature (Tagg) was defined as the temperature at which the antibodies start to aggregate. The melting temperature (Tm) was measured by FluoItensity of the intrinsic light (Tm1). Samples were measured as technical duplicates (n=1).

| | **Tagg [°C]** | **Tm1 [°C]** |
|---|---|---|
| mαCD3 IgG | 65.5 ± 1 | 58.5 ± 1 |
| Prot-mαCD3 IgG | 59.5 ± 1 | 64 ± 1 |
| FOLR1-TCB | 58.5 ± 1 | 63.5 ± 1 |
| Prot-FOLR1-TCB, matA site | 58.5 ± 1 | 60.5 ± 1 |
| Prot-FOLR1-TCB, MMP-matA site | 58 ± 1 | 59.5 ± 1 |
| Prot-FOLR1-TCB, non-cleav | 58.5 ± 1 | 60.5 ± 1 |
| Prot-FOLR1-TCB, MMP site | 61.5 ± 1 | 63 ± 1 |

| | |
|---|---|
| Tagg: | Scatter 266nm |
| Tm1: | FluoIntensity |

## Supplementary Methods

### *Generation of anti-idiotypic anti-CD3 antibodies*

Anti-idiotypic anti-CD3 antibodies were generated by hybridoma technology. Briefly, female NMRI mice were primarily immunized intraperitoneally (i.p.) with 100 µg of the F(ab`)2 fragment of the humanized anti-CD3ε binder formulated in CFA (Complete Freund's Adjuvant). Mice were boosted six and ten weeks after the initial immunization by the intra peritoneal administration of 100 µg F(ab`)2 fragment of the humanized anti-CD3 antibody formulated in IFA (Incomplete Freund's Adjuvant). Subsequently, mice were boosted by two intravenous administrations of 50 µg F(ab`)2 fragment of the anti-CD3 antibody three and two days before the splenocytes of the immunized animals were used for the generation of hybridoma by fusion with P3x63-Ag8.653 myeloma cells. After fusion of spleen cells and screening for anti-idiotypic anti-CD3 binders, RNA from hybridoma cells was prepared using the RNeasy®Mini (Qiagen). Sequences were obtained by RACE-PCR (rapid amplification of cDNA ends) using a GeneRacer Kit (Invitrogen). The following primers were used for anti-idiotypic anti-CD3 4.15 Generacer 5`nested Primer 5′-GGACACTGACATGGACTGAAGGAGTA-3′ and for VL GAB2878 GGGAAGATGGATCCAGTTGGTGCAGCATCAGC and for VH GAB3262 aaatarcccttgaccaggcatcc. The following primers were used for anti-idiotypic anti-CD3 4.32 Generacer 5`nested primer 5′-GGACACTGACATGGACTGAAGGAGTA-3′ and for VL GAB2878 GGGAAGATGGATCCAGTTGGTGCAGCATCAGC and for VH GAB3262 aaatarcccttgaccaggcatcc.

### *Kinetic screening of anti-idiotypic anti-CD3 antibodies*

The anti-idiotypic anti-CD3 antibody was chemically immobilized on a CM5 chip following the manufacturer instructions (Biacore T200, GE Healthcare). The Fab fragment of the anti-CD3 antibody (generated by plasmin digestion and purification) or the FOLR1-TCB were used as the analyte (Supplementary Figure 8) and injected for 180 s in a range of concentrations between 0.23 and 500 nM or 1.83 and 4000 nM for the Fab fragment or the TCBs respectively. Dissociation was monitored for 10 min. Double referencing was applied and fitting with a one-to-one Langmuir binding model provided the kinetic constants of the interaction. Two independent dilution series of analytes were analyzed in the same run and gave the same values.

***Expression, Purification and Quality control***

All antibodies (unless commercially available) were produced in transiently transfected HEK293 cells growing in suspension. The cells were transfected with the corresponding mammalian expression vectors in appropriate ratios. After cultivation supernatant was harvested by centrifugation, sodium azide was added to a final concentration of 0.01 % w/v before the solution was sterile filtered (0.22 µm filter). The solution was kept at 4 °C until purification. The secreted protein was purified by ProteinA affinity chromatography, followed by size exclusion chromatographic steps. The protein concentration of purified protein samples was determined by measuring the optical density (OD) at 280 nm divided by the molar extinction coefficient calculated on the basis of the amino acid sequence. Purity and molecular weight of the molecules after the final purification step were analyzed by CE - SDS in the presence or absence of a reducing agent, using the Caliper LabChip GXII system (Caliper Lifescience) according to the manufacturer's recommendation. The aggregate content of the molecules was analyzed using a TSKgel G3000 SW XL analytical size-exclusion column

(Tosoh) in 25 mM $K_2HPO_4$, 125 mM NaCl, 200 mM L-arginine monohydrocloride, 0.02 % (w/v) $NaN_3$, pH 6.7 running buffer at 25 °C.

***Negative Stain - Transmission Electron Microscopy***

For Negative Stain - Transmission Electron Microscopy (NS-TEM) , stock solutions were diluted to about 4 µg/ml with D-PBS (Gibco Life Technologies). 4 µl of diluted samples were adsorbed for 60 s to glow-discharged parlodion carbon-coated copper grids. The grids were then blotted, washed on five drops of double-distilled water and negatively stained on two droplets of 2 % uranyl acetate (pH 4.3) solution. For complex formation 5 µl of unmasked antibody fragment (about 88 pmoles) were mixed with 7 µl of the Fc-CD3εδ ligand (about 100 pmoles) after 1 hour incubation at room temperature, the mixture was further diluted 300 x with D-PBS for TEM analysis. Samples were imaged at a nominal magnification of 130000 X using a Philips CM100 electron microscope (Philips, Eindhoven, The Netherlands) operating at 80 kV. Electron micrographs were recorded on a 2000 by 2000 pixel charge-coupled device camera (Veleta, Olympus soft imaging solutions GmbH, Münster, Germany) mounted in the 35 mm port of the TEM, yielding a final pixel size of 0.37 nm on the specimen level. Reference-free alignment was performed on manually selected particles from recorded image using the EMAN2 image processing package [2]. A total of 1000 to 2500 particles of each imaged samples were extracted from the micrographs, aligned, and classified by multivariate statistical analysis yielding class averages. The class averages with the best signal-to-noise ratio were selected and presented. The presented class averages result from the average of 60 to 140 single particles. The length of the globular structures were measured on several class averages along their longest dimensions with the Freehand Line toll of the ImageJ1 software [3].

***Atomic force microscopy (AFM)***

A circular mica disc (V1, PLANO GmbH, Wetzlar, Germany) with a diameter d = 10 mm was mechanically cleaved with tweezers. A freshly exposed layer of mica was incubated with the

"Prot-mαCD3 IgG/masked molecule", a Volume V = 60 µl of a concentration c = 0.3 µg/ml in Dulbecco`s Phosphate Buffered Saline (DPBS, Gibco) was deposited. After 1.5 minutes the incubating solution was removed, the disc was blotted off with a filter paper (Richardson, PLANO, Wetzlar, Germany). A thin film remained on the surface and kept the entire surface wet and hydrated. The surface was washed three times by consecutive deposition and removal of imaging buffer, citric acid/sodium hydroxide buffer, approx. 20 mM, at pH = 5 (Certipur, Merck, Darmstadt, Germany), each droplet had a volume of V = 60 µl. A fourth droplet was deposited to remain on the surface, the sample was mounted on the motorized sample stage of Atomic Force Microscope (Dimension ICON, Nanobruker, Santa Barbara, United States).

An ultrasharp AFM cantilever (Hi`Res-C19/Cr-Au, µ-masch, Schaefer-Tec, Switzerland) with a nominal tip radius of r < 1 nm, a nominal resonance frequency ϖ ~ 65 kHz in air (and approx. 28 kHz in the imaging buffer), a nominal force constant f = 0.5 N/m, was mounted on a cantilever holder ("direct drive liquid cantilever holder", Nanobruker, Santa Barbara, United States). To minimize risk of tip damage during tip approach settings as they are established for sharp tips and hard cantilevers in air have been used (www.spmtips.com/how-to-choose-afm-probes-by-resolution-highresolution.html). After single masked molecules had been measured the scanning process was interrupted by lifting the cantilever 3.5 µm above the mica surface. A volume V = 2 µl which contained 2 % of the provided matriptase stock (R&D Systems, 3946-SE-010) solution in PBS was injected. After 5 minutes the raster scanning process was continued, and the same region and molecules were inspected for morphologic changes. After 30 minutes a volume V = 10 µl which contained 1.5 µg/ml of the Fc-CD3 in imaging buffer was injected into the imaging solution. After 5 minutes the scanning process was continued, and the same region and molecules were re-inspected for morphologic changes. All images

were plane corrected using first order plane flattening and median filtering (3x3). The processed images were saved and analyzed with Scanning Probe Image Processor Vers 6.5.1 (Image Metrology).

***CD3 activation assay using Jurkat-NFAT reporter assay***

A Jurkat-NFAT reporter cell line (Promega) (luciferase under control of the NFAT promotor), was used to evaluate activation via CD3 cross-linking. A 96-well white walled clear bottom plate (Greiner BioOne) was coated with 25 µl / well of 8 µg / ml anti-human Fc antibody (BioLegends) in DPBS. The plate was incubated overnight at 4 °C before the DPBS was removed. Cleavage of the Prot-mαCD3 IgG was performed by addition of 1µl of purified recombinant human matriptase (0.44 mg/ml, R&D Systems) at a concentration of approx. 10 nmol of antibody in histidine buffer (20 mmol/l, pH 6, Bichsel) and incubated for 24 hours at 37 °C. The antibodies were diluted in DPBS and added to the coated wells. After incubation for 30 min at 4 °C, the DPBS was removed. The Jurkat-NFAT reporter cells were harvested and viability was assessed using a ViCell device before the cells were resuspended in Jurkat medium (RPMI1640, 2g/l Glucose, 2 g/l $NaHCO_3$, 10 % FCS, 25 mM HEPES, 2 mM L-Glutamin, 1 x NEAA, 1 x Sodium-pyruvate) without Hygromycine. 25,000 Jurkat NFAT-cells / well were added prior to incubation for 5 h at 37 °C in a humidified incubator. The antibodies for the non-coated control were diluted in assay medium and added immediately after the Jurkat NFAT-cells had been added. The plates were taken out of the incubator for approx. 10 min to adapt to room temperature before ONE-Glo substrate solution (1:4 diluted, 25 µl in 75 µl) was added to the wells and incubated for 10 min at room temperature in the dark. Luminescence was detected using WALLAC Victor3 ELISA reader (PerkinElmer2030), with 5 sec / well as the detection time.

***CD3 activation assay using PBMCs as effector cells***

Human PBMCs of three different healthy donors were used as effector cells and T-cell activation was analyzed after 48 h of incubation with the molecules. About 24 h before the assay start, PBMCs were thawed in RPMI1640 medium (10 % FCS, 1 X GlutaMax). The PBMCs were centrifuged at 350 g for 7 min and resuspended in fresh medium (RPMI1640, 10% FCS, 1X GlutaMax). PBMCs were cultured for 20 h to 24 h before they were used in assays. Plates were coated and monovalent CD3 IgGs bound as described above. Cleavage of the Prot-mαCD3 IgG was performed as described above. PBMCs were harvested and viability was assessed using a ViCell device. Cells were resuspended in 50 % fresh medium (RPMI1640, 10% FCS, 1x GlutaMax) and 120 µl per well (100,000 cells / well) were added. The antibodies for the non-coated controls were diluted in assay medium and just added after the PBMCs were added. Cells were incubated for 48 h at 37 °C in a humidified incubator before T-cell activation was analyzed by CD69 quantification on CD8 positive cells [4]. T-cell activation was assessed by quantification CD69 for CD8 positive T-cells using Flourophore labelled antibodies (APC anti human CD8 (BioLegend, 344722), PE-anti human CD69 (BioLegend, 310906), PE-anti human CD8 (BioLegend, 344706)) and a MACSQuant device (Miltenyi Biotec). Data were analyzed by FlowJo V10 software and GraphPad Prism 7 Software.

***Thermal stability of protease-activated TCB***

To determine the thermal stability of the Prot-FOLR1-TCBs containing a matriptase, MMP-2, -9 or combined MMP-2, -9-matriptase linker samples were analyzed using an Optim2 device (Unchained Labs). Samples were prepared at 1 mg / mL in 20 mM histidine chloride, 140 mM NaCl, pH 6.0. Temperature ranged from 25 °C to 85 °C with a heating rate of 0.1 °C / minute. The aggregation onset temperature (Tagg) was defined as the temperature at which the

molecules start to aggregate. The melting temperature (Tm) was analyzed by measurement of FluoIntensity of the intrinsic light (Tm1).

***Serum stability of protease-activated TCB***

Serum stability of the Prot-FOLR1-TCB and the FOLR1-TCB was checked in human IgG depleted serum. The TCBs were incubated for 14 days in serum or buffer at 37 °C. Molecules were purified via affinity chromatography using HPLC before the integrity (capillary electrophoresis at non-reducing conditions) was analyzed.

***Linker cleavage by different recombinant proteases***

A Jurkat-NFAT reporter cell line (Promega) (luciferase under control of the NFAT promotor), was used to evaluate linker cleavage by different proteases via CD3 binding. The Prot-FOLR1-TCBs (130 nM) were incubated with rhMMP-2 (R&D Systems), rhMMP-9 (R&D Systems) or rhMatriptase/ST14 (R&D Systems) for 20 h at 37 °C and subsequently bound to plate coated anti human Fc antibody (providing cross-linking required for T-cell activation). 25,000 Jurkat NFAT-cells / well were added prior to incubation for 5 h at 37 °C in a humidified incubator. Luminescence was detected after addition of One-Glo substrate (Promega) using WALLAC Victor3 ELISA reader (PerkinElmer2030), with 5 sec / well as the detection time.

***Quantification of FOLR1 expression levels***

The QIFIKIT® (Dako) was used for the quantification of FOLR1 expression levels using an anti-human FolR1 (mouse IgG1, LifeSpan BioSciences Inc) and a mouse IgG1 isotype antibody (BD) as control. The median fluorescence intensity of the cells stained with the isotype control was subtracted from the median fluorescence intensity of the cells stained with the anti-FOLR1 antibody before the antigen binding sites were calculated from the standard.

## Supplementary References


1 Desnoyers, L. R. *et al.* Tumor-specific activation of an EGFR-targeting probody enhances therapeutic index. *Sci Transl Med* **5**, 207ra144, doi:10.1126/scitranslmed.3006682 (2013).

2 Ludtke, S. J., Baldwin, P. R. & Chiu, W. EMAN: semiautomated software for high-resolution single-particle reconstructions. *J Struct Biol* **128**, 82-97, doi:10.1006/jsbi.1999.4174 (1999).

3 Schneider, C. A., Rasband, W. S. & Eliceiri, K. W. NIH Image to ImageJ: 25 years of image analysis. *Nat Methods* **9**, 671-675 (2012).

4 Simms, P. E. & Ellis, T. M. Utility of flow cytometric detection of CD69 expression as a rapid method for determining poly- and oligoclonal lymphocyte activation. *Clin Diagn Lab Immunol* **3**, 301-304 (1996).
