# Supplementary material for: Protease-activation using anti-idiotypic masks enables tumor specificity of a folate receptor 1-T cell bispecific antibody
Source: Nat Commun. 2020 Jun 24;11:3196. doi: 10.1038/s41467-020-16838-w (PMC7314773; doi:10.1038/s41467-020-16838-w)
Supplement: Supplementary file 3 — Reporting Summary [file 41467_2020_16838_MOESM3_ESM.pdf]

nature research

Corresponding author(s): Christian Klein, Roche Glycart AG, Wagistrasse 10, CH-8952 Schlieren, Switzerland. Phone: +41 43 215 1628; E-mail: christian.klein.ck1@roche.com

Life Sciences Reporting Summary

Nature Research wishes to improve the reproducibility of the work that we publish. This form is intended for publication with all accepted life science papers and provides structure for consistency and transparency in reporting. Every life science submission will use this form; some list items might not apply to an individual manuscript, but all fields must be completed for clarity.

For further information on the points included in this form, see Reporting Life Sciences Research. For further information on Nature Research policies, including our data availability policy, see Authors & Referees and the Editorial Policy Checklist.

Please do not complete any field with "not applicable" or n/a. Refer to the help text for what text to use if an item is not relevant to your study. For final submission: please carefully check your responses for accuracy; you will not be able to make changes later.

~ Experimental design

1. Sample size

Describe how sample size was determined.

The in vitro killing assays are well established with different TCBs, so we performed technical triplicates and assays were done for three different human PBMC donors.

In vivo efficacy: A number of 9 animals was assigned per group. No statistical methods were used to predetermine the total number of animals needed for this study, however, taking into consideration the heterogeneity of tumors growth as well the heterogeneous humanization rate of NSG mice we experience 9 mice group as a good number for statistical power

2. Data exclusions

Describe any data exclusions.

Fig 7 FOLR1 negative tumor samples (3/5); Fig 9A One outlier in terms of tumor growth has been excluded from the analysis. This mouse seemed to lose the humanization over the course of the study which explained the poor response.

3. Replication

Describe the measures taken to verify the reproducibility of the experimental findings.

The in vitro killing assays are well established with different TCBs, so we performed technical triplicates and assays were done for three different human PBMC donors.
For in vivo efficacy study 9 animals were assigned per group. The study was conducted once. To account for variations we have used a high sample size per treatment group, we clearly indicate the error bars in the tumor growth curves and indicate statistical measures.

4. Randomization

Describe how samples/organisms/participants were allocated into experimental groups.

Human PBMCs were randomly allocated for the assays. Tumor cell lines were harvested and seeded to wells for different treatments.
Mouse allocation to vehicle and treatment groups was driven by 3 parameters: body weight, tumor volume and human CD3+ T cell counts in blood. A Roche internal randomization tool has been developed by statisticians that performs unbiased automated randomization of animals by using the variables mentioned above and defined cut-offs for each of those (e.g. all tumors bigger than 500mm3 at the day randomization are excluded)

5. Blinding

Describe whether the investigators were blinded to group allocation during data collection and/or analysis.

In vitro: Investigators were not blinded. The Antibodies are labeled with IDs, however everyone could track back which antibody is in the vial. Blinding is therefore not feasible with the work situation.
In vivo: The investigators were not blinded during group allocation or data collection, however, as described above, group allocation was done by an unbiased automated randomization tool. People that organize and plan in vivo studies are not the same that eventually get the drugs to inject. The antibodies are labeled with IDs, that could be tracked back by the operators. Blinding is therefore not feasible with the work situation.

Note: all in vivo studies must report how sample size was determined and whether blinding and randomization were used.

6. Statistical parameters

For all figures and tables that use statistical methods, confirm that the following items are present in relevant figure legends (or in the Methods section if additional space is needed).

n/a	Confirmed	
☐	☒	The exact sample size (*n*) for each experimental group/condition, given as a discrete number and unit of measurement (animals, litters, cultures, etc.)
☐	☒	A description of how samples were collected, noting whether measurements were taken from distinct samples or whether the same sample was measured repeatedly
☐	☒	A statement indicating how many times each experiment was replicated
☐	☒	The statistical test(s) used and whether they are one- or two-sided<br>*Only common tests should be described solely by name; describe more complex techniques in the Methods section.*
☐	☒	A description of any assumptions or corrections, such as an adjustment for multiple comparisons
☐	☒	Test values indicating whether an effect is present<br>*Provide confidence intervals or give results of significance tests (e.g. P values) as exact values whenever appropriate and with effect sizes noted.*
☐	☒	A clear description of statistics including central tendency (e.g. median, mean) and variation (e.g. standard deviation, interquartile range)
☐	☒	Clearly defined error bars in all relevant figure captions (with explicit mention of central tendency and variation)

See the web collection on statistics for biologists for further resources and guidance.

~ Software

Policy information about availability of computer code

7. Software

Describe the software used to analyze the data in this study.	FlowJo V10; LabCip GX; GraphPad Prism 7; Biovia Discovery Studio 17R2; GIMP 2.1; Sanning Probe Image Processor Vers 6.5.1; Definiens Developer XD version 2.7; JMP version 12 (SAS Institute Inc., Cary, NC)

For manuscripts utilizing custom algorithms or software that are central to the paper but not yet described in the published literature, software must be made available to editors and reviewers upon request. We strongly encourage code deposition in a community repository (e.g. GitHub). *Nature Methods* guidance for providing algorithms and software for publication provides further information on this topic.

~ Materials and reagents

Policy information about availability of materials

8. Materials availability

Indicate whether there are restrictions on availability of unique materials or if these materials are only available for distribution by a third party.	Material might be obtained under Material transfer agreement (MTA)

9. Antibodies

Describe the antibodies used and how they were validated for use in the system under study (i.e. assay and species).	APC anti human CD8 (BioLegend, 344722) 0.8µl/well (in 25µl with 0.2mio PBMCs), QC and references described here https://www.biolegend.com/de-de/products/apc-anti-human-cd8-antibody-6531;<br><br>PE-anti human CD69 (BioLegend, 310906) 0.8µl/well (in 25µl with 0.2mio PBMCs), QC and references described here https://www.biolegend.com/en-us/products/pe-anti-human-cd69-antibody-1672, T cells incubated with tumor cells but without treatment were used as controls.<br><br>PE-anti human CD8 (BioLegend, 344706) 0.8µl/well (in 25µl with 0.2mio PBMCs), QC and references described here https://www.biolegend.com/en-us/products/pe-anti-human-cd8-antibody-6247<br><br>Purified anti-human IgG Fc Antibody (409302, BioLegend) 8µg/ml, QC and references described here https://www.biolegend.com/fr-ch/products/purified-anti-human-igg-fc-7129<br><br>IHC antibodies:<br>anti-human ST-14 (PA5-29764 from Thermo Scientific, Germany);<br>human folate receptor alpha with anti-FOLR1 (BN3.2, Byosystems, Switzerland);<br>human T cell detection with anti-CD3 (ab5690, Abcam, Germany)<br>FOLR1 and CD3 dilution=1/100 and ST-14 dilution =1/600<br>The antibodies were selected with the following criteria: the most common used in the literature published in high impact journals and provided by reliable commercial vendors. Furthermore we validated/ evaluated the antibodies in house for specificity, sensitivity and reproducibility utilizing isotype control antibodies, negative stainings without the primary antibodies and testing positive and negative tissues in an automated platform. All antibodies were titrated for optimal dilution before the final assay.

10. Eukaryotic cell lines

a. State the source of each eukaryotic cell line used.	HEK 293 cells, HeLa and Skov-3 cells were purchased from ATCC (American Type Culture Collection), human bronchial epithelial cells (HBEpiC, 3210) and human renal cortical epithelial cells (HrcEpiC, 4110) were purchased from ScienCell Research Laboratories, AsPC-1 (ECACC, 96020930) cells were obtained from the European Collection of Cell Cultures (ECACC) and NCI H596 cells were provided by Roche Innovation Center Munich. MDA-MB-231 NLR were purchased from EssenBioscience (Cat.# 4487). Jurkat NFAT-cells were purchased from Promega.
b. Describe the method of cell line authentication used.	Cell identity of all tumor cell lines was verified by LGC cell line authentication service provided by American Type Culture Collection (ATCC) . Cells were harvested and counted. The cells were washed with DPBS and finally spotted on paper provided with the kit. ATCC performs STR Profiling following ISO 9001:2008 and ISO/IEC 17025:2005 quality standards.
c. Report whether the cell lines were tested for mycoplasma contamination.	All tumor cell lines were routinely tested for mycoplasma contamination. All tumor cell lines used herein were tested negative for mycoplasma contamination.
d. If any of the cell lines used are listed in the database of commonly misidentified cell lines maintained by ICLAC, provide a scientific rationale for their use.	Cell identity of all tumor cell lines was verified by cell authentication service provided by American Type Culture Collection (ATCC).<br>None of the cell lines used for in vitro assays was listed in ICLAC database of commonly misidentified cell lines.

Animals and human research participants

Policy information about studies involving animals; when reporting animal research, follow the ARRIVE guidelines

11. Description of research animals

Provide all relevant details on animals and/or animal-derived materials used in the study.

4–5 week old female NOD scid gamma (NSG) mice were used in the described study.
Humanized mice were generated as described in the in vivo analysis section: Hematopoietic stem cell humanized mice (humanized mice), used for efficacy or single dose PK studies, were generated in house. Briefly, 4–5 week old female NOD scid gamma (NSG) mice (Jackson Laboratory, Sacramento, CA USA) were injected i.p. with 15 mg/kg Busulfan (Busilvex, Pierre Fabre Limited) in a total volume of 200 µL. Twenty-four hours later, mice were injected intravenously (i.v.) with 1 × 105 CD34+ cord blood cells (STEMCELL Technologies Inc, Grenoble, France). Fifteen weeks after cell injection, mice were bled and screened for successful humanization by flow cytometry.

The in vivo efficacy study was carried out in an AAALAC accredited Animal Facility. All animal studies were performed in accordance with the Federation for Laboratory Animal Science Associations (FELASA). The mice were housed in groups of five animals per cage (Type M3, 820 cm^2) and had access to autoclaved water and pelleted feed. The cage environment was enriched with a mouse house, nesting material and a sterile wooden stick. The mice were kept at a standard temperature of 22°C±2°C and a relative humidity of 55% (45-70%) in a 12:12-hour light:dark cycle (lights on, 6 am to 6 pm). The animal studies were approved by and done under license from the Government of Upper Bavaria (Regierung von Oberbayern; Approval number: ROB-55.2-2532.Vet_03-16-10)
The SDPK in vivo studies in non-tumor bearing mice were carried out in an AAALAC accredited Animal Facility. Animals were acclimatized for 7 days before the start of the experiment. The mice were housed in individually ventilated cages (Green Line, Tecniplast, Italy) and had access to acidified and autoclaved water and sterile pelleted feed (Granovit AG, Kaiseraugst, Switzerland) ad libitum. The bedding was a standard woodchip bedding Granovit AG, Kaiseraugst, Switzerland) and the cage environment was enriched with a mouse house (Indulab, Switzerland), nesting material and a sterile wooden stick. The mice were kept at a standard temperature of 22°C±2°C and a relative humidity of 55%±10%, in a 12:12-hour light:dark cycle (lights on, 7 am to 7 pm) at a light range intensity of 130 - 300 lux. All experimental protocols were approved by the Cantonal Veterinary Authority of the Canton of Zurich and were conducted in accordance with the Swiss Animal Welfare legislation.

Policy information about studies involving human research participants

12. Description of human research participants

Describe the covariate-relevant population characteristics of the human research participants.

Human PBMCs from healthy donors were isolated from buffy coats obtained from Blutspende Zürich SRK. The Blutspende Zürich SRK confirmed to us that all donors consented into the use of the sample for research purpose.
